# Discharge against Medical Advice in Surgical Patients with Posttraumatic Stress Disorder: A Case Report Series Illustrating Unique Challenges

**DOI:** 10.1155/2017/3045907

**Published:** 2017-06-21

**Authors:** Marek Brzezinski, Maren Gregersen, Luiz Gustavo Schuch, Ricarda Sawatzki, Joy W. Chen, Grant Gauger, Jasleen Kukreja, Brian Cason

**Affiliations:** ^1^VA Medical Center, Anesthesia Services, 4150 Clement Street, San Francisco, CA 94121, USA; ^2^Department of Anesthesia and Perioperative Care, University of California, San Francisco, 521 Parnassus Avenue Room C-450, San Francisco, CA 94143-0648, USA; ^3^VA Medical Center, Neurosurgery Services, 4150 Clement Street, San Francisco, CA 94121, USA; ^4^Department of Neurosurgery, University of California, San Francisco, 400 Parnassus Ave., San Francisco, CA 94143, USA; ^5^VA Medical Center, Surgery Services, 4150 Clement Street, San Francisco, CA 94121, USA; ^6^Department of Surgery, University of California, San Francisco, 400 Parnassus Ave., San Francisco, CA 94143, USA

## Abstract

Discharge against medical advice (DAMA) can have detrimental effects on patient outcomes. Recently, the diagnosis of posttraumatic stress disorder (PTSD) has been linked with DAMA in the* mental health* setting. However, PTSD as a risk factor for DAMA in* surgical* patients has not received much consideration, although such patients may be at risk for triggering or amplification of PTSD symptoms perioperatively. We present the first case report series of three surgical patients with PTSD who left the hospital AMA. These cases differ markedly from DAMA in non-PTSD patients. In all three subjects, the stress of feeling misunderstood by clinicians and the distress of public detainment by hospital security in the setting of chronic PTSD led to aggressive and risky behavior. All three subjects represented a risk to themselves and to others at the time of DAMA. Finally, all three subjects were difficult to contact for follow-up or medical care and missed appointments.

## 1. Introduction

Up to 2% of inpatients in the US leave the hospital against medical advice (AMA) [1, 2]. Discharge AMA (DAMA) has been associated with poor compliance, increased mortality and morbidity, higher readmission rates, longer subsequent hospital stays, increased health care costs, and increased risk of suicide [1, 2]. Furthermore, DAMA can lead to long-term patient stigmatization in health care settings, compounding the aforementioned problems [1].

Psychiatric comorbidities are frequently associated with DAMA [3, 4]. The rate of DAMA in psychiatric patients in mental health settings has been reported to be between 6% and 35% [4]. Recently, diagnosis of posttraumatic stress disorder (PTSD) in* psychiatric hospitalized* patients was reported to be associated with hostile behavior at the time of discharge and increased likelihood of DAMA [5]. PTSD as a risk factor for DAMA in* surgical* patients, however, has not received much consideration, although* surgical patients with PTSD* may be at particularly high risk for triggering or amplifying PTSD symptoms as a result of perioperative pain and stress. Disconcertingly, DAMA could have more detrimental sequelae in patients with PTSD, as they are already at high risk for poor compliance, loss to follow-up, suicide, and increased health care costs [6–9].

Herein, we present three surgical patients with PTSD diagnosis who left AMA, focusing on patient behavior and challenges to health care providers. These cases differ markedly from DAMA in non-PTSD patients. With steadily increasing numbers of patients with PTSD requiring surgical procedures, health care providers must be familiar with the unique challenges these patients present in the perioperative period.

## 2. Case Reports

### 2.1. Case 1

A 61-year-old man underwent placement of a left superficial femoral artery stent without complications. His past medical history included war-related PTSD, diabetes, hypertension, and heavy smoking. Medications included aspirin, lisinopril, glyburide, metformin, and fentanyl patch. Shortly after his surgical procedure, the patient threatened to leave AMA unless he was allowed to* “roll around”* in a wheelchair* “as he pleased*.*”* He was permitted to briefly leave the ward with his peripheral intravenous (IV) catheter left in place. The next morning, the patient was awake, alert, and fully oriented. About two hours later, he was increasingly lethargic with apneic episodes and oxygen saturation 80–85%. Other vital signs were unremarkable. His glucose was 246 mg/dL and was treated accordingly. After he was given Narcan (0.2 mg IV), the patient became markedly more alert and was transferred to the Intensive Care Unit (ICU). He denied self-administration of medication. A urine toxicology test was positive for benzodiazepines (received during anesthesia the day before). His arterial blood gases demonstrated a hypercarbic respiratory acidosis with pH 7.32, P_a_CO_2_ 68.3 mmHg, and P_a_O_2_ 99 mmHg. After he received a second dose of Narcan (0.2 mg), he became agitated and wheeled himself out of the unit and eventually the hospital with IV still in place and his care team in pursuit. He got in his car and drove until he encountered a barricade set up by hospital police officers to stop him as he presented a potential danger to himself and to others. The patient agreed to return to the ICU but refused all tests. The next morning, despite his systolic blood pressure being 231 mmHg, the patient left the hospital AMA after removal of his peripheral IV catheter. He never returned to our facility for follow-up or medical care.

### 2.2. Case 2

A 60-year-old man underwent an elective video-assisted thoracoscopic surgery for lung biopsy. His past medical history was significant for heavy smoking with chronic dyspnea and war-related PTSD. His medications included inhalers and prazosin. His surgery and postoperative course were unremarkable. Around 5:00 a.m. on the morning of postoperative day 2, the patient declared that he would be leaving the hospital at 7:00 a.m. and demanded removal of the peripheral IV catheter. Upon being informed that the removal can only occur with a written order by his doctor, the patient left the ward. The grounds were searched but he was unable to be located. Shortly thereafter, the patient returned to the unit and stated,* “I want this IV out and I am leaving at 0700*.*”* After the risks associated with leaving AMA were discussed (e.g., tension pneumothorax) and understood by the patient, his peripheral IV was removed, and he left the hospital AMA for a long distance drive home alone. Subsequently, he missed follow-up appointments and refused emergency treatment when he developed cardiac chest pain at home (unrelated to surgery).

### 2.3. Case 3

A 57-year-old man was admitted to the hospital after being found at home lethargic and minimally responsive after a fall. The noncontrast computer tomography (CT) of the head showed a large, acute-on-chronic right subdural hematoma measuring up to 1.7 cm, with a 6 mm right-to-left midline shift and subfalcine herniation. The patient was admitted to ICU for assessment and urgent treatment. His past medical history was significant for diabetes, polysubstance abuse, war-related PTSD, and heavy smoking. The patient had a history of noncompliance. His medications on admission included metformin, trazodone, and methadone. Given the clinical presentation and head-CT findings, the neurosurgical team recommended proceeding emergently with evacuation of the subdural hematoma. However, the patient refused to consent stating that he would rather die than have surgery, stating* “It would be fine [if he died]; it would relieve me of my pain”*, but that he* “will not die,” *because he was a Navy Seal. Psychiatric consultation found the patient lacking in decision-making capacity. Given the danger to self and the potential threat he posed to others, they recommended holding the patient in the ICU and restarting his home medications. Psychiatric reassessment a day later determined that the patient had regained his decision-making capacity. His vital signs, mental status, and neurological exam results were stable. Following thorough presentation and discussion of the risks associated with leaving AMA and assertion by the patient that he understood, he left the hospital. Subsequently, he was difficult to contact and missed appointments.

## 3. Discussion

DAMA represents a well-recognized problem with approximately 500,000 cases estimated every year in the US [1, 2] and results in adverse patient outcomes [1, 13–15]. Patients leaving AMA have higher mortality and hospital readmission rate, increased risk of suicide, and higher overall healthcare costs [1, 3, 16]. Moreover, DAMA is likely to impair the doctor-patient relationship and reduce patient adherence to medical treatments [1, 2]. Known predictors of DAMA include male gender, younger age, lower socioeconomic class, history of substance abuse, lack of health insurance, and previous history of leaving AMA [1, 3, 10, 11, 17, 18].

Recently, the diagnosis of PTSD was shown to be associated with hostile behavior at hospital discharge and more than a sixfold increase in risk of DAMA in the mental health setting [5]. The lifetime prevalence of PTSD is estimated to be 8% among US citizens [6] and 10–18% among military veterans [7], yet PTSD as a risk factor for leaving AMA in surgical inpatients has not received much consideration. PTSD [6, 8, 9, 19] and DAMA [1, 13, 14] are independently associated with poor compliance, loss to follow-up, suicide, and high costs of health care. Some aspects of PTSD, such as physical and cognitive deficits, behavioral issues, and anger and aggression problems, can interfere with decision-making abilities [1]. Therefore, DAMA in surgical patients with PTSD could amplify the negative effects leading to worse outcome than in patients without PTSD [6–9]. We present the first case series of DAMA demonstrating unique challenges in surgical patients with PTSD who left AMA either before or after scheduled surgery.

Caring for such patients can be challenging. Aggression at the time of discharge was expressed by all three patients and required the use of hospital security. Such interactions negatively impact the patient-doctor relationship and alienate the patient. Conversely, aggressive behavior by the patient could also distress the health care providers and damage their perception of the patient or potentially any patient with PTSD, compounding the problem further.

Such patients pose risk to themselves and others. Two patients disappeared from the ward with a functioning peripheral IV catheter in place (Cases 1 and 2). Leaving with an IV in situ poses the risk of self-administration of drugs and driving under the influence. In fact, one patient attempted to drive away until the hospital police intervened.

Stress of attempting to leave the hospital AMA, the perception of being misunderstood, and emotionally charged interactions with staff and police could exacerbate PTSD symptoms. While the severity of PTSD was not explicitly measured at the time of DAMA, at least one patient made comments related to war.

All three patients showed decreased compliance following DAMA and either were difficult to contact, missed appointments, or never returned to our facility for any follow-ups. Abrupt DAMA makes the preparation for future follow-up almost impossible. Decreased compliance has long-term impact on PTSD surgical care and PTSD care, exacerbating PTSD symptoms. The issues summarized above highlight the potential risk of increased long-term health care costs in the surgical population with PTSD.

The link between PTSD and DAMA needs to be examined more rigorously in a perioperative context. Meanwhile, clinicians should focus on establishing strong patient-physician relationship, communicating effectively with the patient, and constructively addressing patient's concerns [1, 2, 10–12, 20]. These interventions should be complemented with PTSD-unique considerations (Table 1) that focus on maintaining the therapeutic bond by ensuring a safe discharge and supporting the patient through follow-ups [1, 2, 10–12].

In conclusion, this series highlights how the acute stress of feeling misunderstood by clinicians and the distress of public detainment by hospital security—in addition to the chronic stress of PTSD—can lead to more aggressive and risky behavior in the perioperative period. Patients with PTSD leaving AMA represent a risk to themselves and to others and may suffer from more negative long-term sequelae. Consequently, DAMA could have more detrimental effects on patients with PTSD than those without. With increasing numbers of surgical patients with preexistent PTSD, health care providers should be familiar with the unique challenges these patients present in the perioperative period.

## Figures and Tables

**Table 1:** Interventions for consideration in surgical patients with PTSD [1, 2, 10–12].

Consideration	Intervention
Be proactive.	Identify history of AMA, poor compliance, or violence to better assess current risk.
	Provide social and psychological support.
	Provide realistic information about the postoperative period.
	Maintain open patient-doctor communication.
	Ensure the continuation of medication for PTSD while being in the hospital.

Be aware and prepared.	Develop collaborative approaches and effective rescue strategies that could be implemented as soon as the patient wishes to leave AMA.
	Handle aggressive and hostile behaviors in a productive and constructive way.
	Avoid getting upset or frustrated; instead, be positive and encouraging.
	Try to reduce the danger of losing the patient's trust by utilizing communication that emphasizes understanding of their unique issues with cultural sensitivity.

Decrease stigma.	Actively maintain the patient-doctor-relationship, ensuring proper follow-up.

*Note*. AMA = against medical advice; PTSD = posttraumatic stress disorder.
